# Cohesive Regulation of Neural Progenitor Development by microRNA miR-26, Its Host Gene Ctdsp and Target Gene Emx2 in the Mouse Embryonic Cerebral Cortex

**DOI:** 10.3389/fnmol.2018.00044

**Published:** 2018-02-19

**Authors:** Haijun Zhang, Longbin Zhang, Tao Sun

**Affiliations:** ^1^Department of Cell and Developmental Biology, Weill Cornell Medical College, Cornell University, New York, NY, United States; ^2^Department of Genetic Medicine, Weill Cornell Medical College, Cornell University, New York, NY, United States; ^3^Center for Precision Medicine, School of Medicine and School of Biomedical Sciences, Huaqiao University, Xiamen, China

**Keywords:** miR-26, Ctdsp, Emx2, neural progenitor, cell-cycle progression

## Abstract

Proper proliferation and differentiation of neural progenitors (NPs) in the developing cerebral cortex are critical for normal brain formation and function. Emerging evidence has shown the importance of microRNAs (miRNAs) in regulating cortical development and the etiology of neurological disorders. Here we show that miR-26 is co-expressed with its host gene *Ctdsp* in the mouse embryonic cortex. We demonstrate that similar to its host gene Ctdsp2, miR-26 positively regulates proliferation of NPs through controlling the cell-cycle progression, by using miR-26 overexpression and sponge approaches. On the contrary, miR-26 target gene Emx2 limits expansion of cortical NPs, and promotes transcription of miR-26 host gene *Ctdsp*. Our study suggests that miR-26, its target Emx2 and its host gene *Ctdsp* cohesively regulate proliferation of NPs during the mouse cortical development.

## Introduction

The precisely regulated proliferation, survival and differentiation of neural stem cells (NSCs) and neural progenitors (NPs) determine normal development of the mammalian cerebral cortex (Dehay and Kennedy, 2007; Rakic, 2007, 2009; Aguirre et al., 2010; Delaunay et al., 2017; Subramanian et al., 2017). During embryonic cortical development, NSCs first appear as the highly proliferative neuroepithelial cells lining the ventricular wall. They subsequently generate radial glial cells (RGCs) or outer radial glia (oRG), which undergo an asymmetric division (Bultje et al., 2009; Wang et al., 2011; Ostrem et al., 2014). RGCs are tightly regulated to maintain their own population while to produce intermediate progenitors (IPs) that reside in the subventricular zone (SVZ) and mature neurons that migrate into the cortical plate (CP; Guillemot, 2005; Kriegstein et al., 2006; Mizutani et al., 2007; Molyneaux et al., 2007; Molnár, 2011; Franco and Müller, 2013). Abnormal proliferation or/and differentiation, caused by prenatal or postnatal gene mutations and altered gene expression levels, is one of the critical factors for brain malformation (Chenn and Walsh, 2002; Piao et al., 2004; Sun and Hevner, 2014). Molecular mechanisms that regulate expression patterns and levels of critical genes that govern cortical development remain an exciting research topic.

The emerging evidence indicates essential roles of microRNAs (miRNAs) in diverse biological events, such as cell proliferation, differentiation, migration, apoptosis and tumorigenesis (Kremer-Tal et al., 2004; Bian and Sun, 2011; Nadim et al., 2017). miRNAs are ~22 nucleotide (nt) endogenous noncoding small RNAs acting in complex gene regulatory networks (Lee et al., 1993; Wightman et al., 1993; Cora et al., 2017). Mature miRNAs recognize a complementary sequence frequently in the 3′-untranslated region (3′UTR) of its target messenger RNA (mRNA) to affect mRNA stability and/or silence protein translation (Carthew and Sontheimer, 2009; Kim et al., 2009). Studies have shown that misregulation of miRNAs generated by cortical *Dicer* deletion causes significant cell death, loss of progenitors and abnormal differentiation (De Pietri Tonelli et al., 2008; Kawase-Koga et al., 2009, 2010; Hong et al., 2013). Knockout of specific miRNA has shown a significant impact of miRNAs in embryonic and adult neurogenesis (Shen et al., 2006; Aguirre et al., 2010; Krol et al., 2010; Mellios et al., 2011; Bian et al., 2013; Abdullah et al., 2016). Furthermore, dysfunction of miRNAs is associated with the pathogenesis of neurodevelopmental disorders, neurodegeneration diseases and affective mental disorders (Hugon and Paquet, 2008; Packer et al., 2008; Bian and Sun, 2011).

miRNA miR-26 has previously been described as a functional miRNA that is involved in various biological events such as cell proliferation, development of normal tissues and tumorigenesis (Gao and Liu, 2011). Interestingly, studies have shown a contradictive role of miR-26 as either a tumor suppressor or activator in different types of cancer via regulating cell proliferation and migration (Lu et al., 2011; Zhang et al., 2012; Tan et al., 2014; Du et al., 2015). Further studies have illustrated a regulatory role of miR-26 in G1/S-phase transition by concomitantly expressing with their host genes *C-terminal domain RNA polymerase II small phosphatase* (*Ctdsp* gene families; Zhu et al., 2012; Wang et al., 2016). Despite these reports in tumors, the role of miR-26 in cortical development has not been well explored.

This study demonstrates that miR-26 and its host gene *Ctdsp* are co-expressed in NPs in the mouse developing cortex, and they play a positive role in NP expansion. We show that Emx2 is a target gene of miR-26, and displays an opposing function in NP development, compared to miR-26. Moreover, Emx2 functions as a transcription activator to initiate expression of *Ctdsp*. Our results elucidate a regulatory loop of miR-26, their target gene Emx2 and host gene *Ctdsp*, which works cohesively to ensure proper development of NPs in the developing cortex.

## Materials and Methods

### Plasmid DNA Constructs

The full-length sequence of *Ctdsp2* with flanking regions was cloned from its cDNA and inserted into the backbone plasmids pCAGIG to construct the overexpression vectors of *Ctdsp2*. The *Emx2* overexpressing constructor was achieved in the same strategy. For *Ctdsp2* silencing, the *Ctdsp2* specific short hairpin RNA (sh*Ctdsp2*) targeting 5′-GCCTGTTGAGGCAGCAGAAGC-3′ was cloned into the pSilencer vector. The *Ctdsp2* knockdown efficiency by this vector was verified by real time reverse transcription PCR. The overexpression and knockdown plasmids of Emx2 was constructed as introduced above.

The mouse genomic sequence including miR-26a precursor was amplified by PCR, and cloned into pGEM-T (promega), following subcloned into the pCAGIG vector for *in utero* electroporation and into pcDNA3.1 (Invitrogen) for transfection, respectively. The following primers were used to amplify miR-26a: F-5′-GGACAAGAACCAGGAAGG-3′, and R-5′-GCTGCCTCCGCGTTCGC-3′. For miR-26a mutation construct, the wild-type miR-26a seed sequence 5′-UCAAGU-3′ was mutated to 5′-UGTTCU-3′ following the instruction of the QuikChange II Site-Directed Mutagenesis Kit (Agilent).

To knockdown the expression of miR-26a, miRNA sponge strategy was used according to previous description (Zhang et al., 2013; Pollock et al., 2014). Briefly, synthesis was operated to construct specific miR-26-related sponges, using forward and reverse sponge oligos (mmu-mir-26a-SP-F: 5′-AC TAGTGTTATCAGCCTATCCTGCTTACTTGAAGTTATCAG CCTATCCTGCTTACTTGAAGTTATCAGCCTATCCTGCTT ACTTGAATCTAGA-3′; mmu-mir-26a-SP-mut-F: 5′-ACTAG TGTTATCAGCCTATCCTGCTTAGTTCTAGTTATCAGCCT ATCCTGCTTAGTTCTAGTTATCAGCCTATCCTGCTTAGT TCTATCTAGA-3′) containing three bulged miR-26a, miR-26a with three mutations in the binding seed, or scrambled binding sites. Each miR-26 sponge contained multiple binding sequences complementary to mature miR-26. All sponges were flanked by the SpeI and XbaI cutting sites, and subcloned into 3′UTR of Pol II-driven green fluorescence protein (GFP) reporter gene, following by inserting into the pCBR conditional expression vector.

### *In Situ* Hybridization

*In situ* hybridization for genes expression was performed on frozen sections using specific probes. Probes used in miRNA hybridization contain modified nucleotides that form a locked structure to stabilize LNA/RNA duplex, thus has been widely used to detect miRNA expression (Zhang and Yin, 2005; Elmen et al., 2008). After fixation with 4% paraformaldehyde (PFA), acetylation with acetylation buffer (1.3% Triethanolamine, 0.25% Acetic anhydride, 20 mM HCl), treatment with proteinase K (5 μg/ml, IBI Scientific) and pre-hybridization (1× SSC, 50% Formamide, 0.1 mg/ml Salmon Sperm DNA Solution, 1× Denhart, 5 mM EDTA, pH 7.5), brain sections were hybridized with DIG-labeled LNA probes at Tm-22°C overnight. After washing with pre-cooled wash buffer (1× SSC, 50% Formamide, 0.1% Tween-20) and 1× MABT, sections were blocked with blocking buffer (1× MABT, 2% Blocking solution, 20% heat-inactived sheep serum) and incubated with anti-DIG antibody (1:1500, Roche) at 4°C overnight. Brain sections were washed with 1× MABT and Staining buffer (0.1 M NaCl, 50 mM MgCl_2_, 0.1 M Tris-HCl, pH9.5), stained with BM purple (Roche) at room temperature until ideal intensity was reached. The miR-26 LNA probe was purchased from Exiqon with specific sequence (5′-UUCAAGUAAUCCAGGAUAGGCU-3′), the Ctdsp2 and Emx2 detective probe were reversed from the amplification of each mRNA using specific primer pairs (Ctdsp2: F-5′-TGCCTCCTGCTTCTCGTTAT-3′, R-5′-GGA CCTCGTGTGTGGAAACT-3′; Emx2: F-5′-TAGAGCACGCT TTTGAGAAGAACCA-3′, R-5′-TGAAACCATACTTTTACC TG-3′), respectively. Each probe was 3′- and 5′-end labeled with DIG–ddUTP.

### Transcriptional Profiling of Ctdsp2 and miR-26 Precursors Genes in Cortex

Total RNA was isolated from the cerebral cortex of E12.5, E15.5 and P0 wild-type CD1 mice using the RNeasy Mini kit (Qiagen) according to the guideline’s instructions. All samples were treated with DNase to remove genomic DNA and reversely transcribed into cDNAs using a Random Hexamer primer (Roche). Three cDNA samples (10× dilution) of each cortex were used as templates to quantify the transcript of each *Ctdsp2* or miR-26 precursors via quantitative real-time PCR (qRT-PCR) with paired primers. qRT-PCR was carried out using primers specific for Ctdsp2: 5′-GCATCTTACATCTTCCAC-3′ and 5′-TAGACATCATCAGTTCCA-3′; and universal primer: 5′-GCGAGCACAGAATAAATACGACTC-3′, together with specific primers for miR-26a-1: 5′-CCTATTCTTGGTTACTTG CACG-3′; miR-26a-2: 5′-CCTGTTCTTGATTACTTG TTTC-3′; miR-26b: 5′-TTCAAGTAAT TCAGGATAGGT-3′; and U6: 5′-CGCTTCGGCAGCACATATAC-3′ and 5′-GTGTCATCC TTGCGCAGGG-3′. The U6 was used as an internal standard. Relative transcript level of each gene was calculated as the ratio of its transcript in each RNA samples over that in the E12.5 RNA samples using the 2^−ΔΔCt^ method (Livak and Schmittgen, 2001).

### Northern Blotting of miR-26a

Total RNA was extracted and separated on 15% urea-PAGE gel, followed by transferred to positively charged nylon membrane (PerkinElmer, Waltham, MA, USA) using Trans-Blot SD Semi-Dry transfer Cell (Bio-Rad, Hercules, CA, USA). Oligonucleotide probes used for hybridization with miRNA were labeled with at their 5′ end. U6 was used as the loading control. The sequence of the probe was: miR-26a: 5′-ATTC AAGTTTTGAAACAGGTGTA-3′.

### *In Utero* Electroporation

*In utero* electroporation was performed in E13.5 embryos according to the published protocol (Saito, 2006). Briefly, plasmid DNA was prepared using the EndoFree Plasmid Maxi Kit (Qiagen) according to manufacturer’s instructions, and diluted to 2 μg/μl. DNA solution was injected into the lateral ventricle of the cerebral cortex, and electroporated with five 50-ms pulses at 35V using an ECM830 electrosquareporator (BTX). Embryos were allowed to develop to E14.5. Animals with the brains electroporated, as detected by the GFP fluorescence under a fluorescent dissection scope (Leica, MZ16F), were selected for further analyses.

All experimental procedures involving animals were in accordance with the Guide for the Care and Use of Laboratory Animals (NIH publications Nos. 80-23, revised 1996) and were performed according to the institutional ethical guidelines for animal experiments.

### Luciferase Assays

Mouse Neuro2a cells were transfected using Lipofectamine 2000 (Invitrogen) using the manufacturer’s protocol. Plasmids were quantified by UV spectrophotometry and used for transfection in a 2:1 ratio (miRNA: target luciferase constructs). pGL4.13 firefly luciferase was used for 3′-UTRs of targets. pGL4.73 *Renilla* luciferase (Promega) was used as a transfection control. For transfections, Neuro2a were diluted in DMEM and plated into 24-well plates in triplicate at 1.5 × 10^4^ cells/ 100 μL. Adherent cells were co-transfected with 100 ng/mL luciferase reporter containing the Emx2–3′-UTR and 50 nM pcDNA3.1 only (control), miR-26a mimics or miR-26amut. Each co-transfection was injected into pcDNA-iCre, miR-26a-SP, or miR-26a-SPmut expressing cells, respectively. After 48 h, luciferase was measured using the Dual-Luciferase Reporter Assay kit (Promega) using the manufacturer’s protocol and read on a Victor3 1420 multilabel counter (Perkin Elmer). All conditions were run in triplicate, and all experiments were repeated at least three times with similar results. Raw results for each condition were normalized for transfection efficiency as the ratio of *Firefly* luciferase to *Renilla* luciferase, and finally for each luciferase tested the empty vector control experiment was set to 1 for display.

### Tissue Preparation and Immunohistochemistry

For embryonic immunohistochemistry, the DNA constructs were injected into E13.5 embryos using *in utero* electroporation. To access proliferation of NP cells (NPCs) in developing cortex, one dose of bromodeoxyuridine (BrdU; 50 mg/g body weight) was administrated by intraperitoneal (I.P.) injection to DNA constructs-injected mice at 1 h or 1 day before sacrifice. Mouse embryonic brains were collected and fixed in 4% PFA in phosphate-buffered saline (PBS) over night, or incubated in 25%–30% sucrose in PBS, embedded in OCT and stored at −80°C until use. Brains were sectioned (14–16 μm) at a coronal plane using a cryostat. For antigen recovery, sections were incubated in heated (95–100°C) antigen recovery solution (1 mM EDTA, 5 mM Tris, pH 8.0) for 15–20 min, followed by 20–30 min of cooling treatment at 4°C. After being blocked in 10% normal goat serum (NGS) in PBS with 0.1% Tween-20 (PBT) for 1 h, sections were incubated with primary antibodies at 4°C overnight, then visualized after 1.5-h co-cultured with goat anti-rabbit IgG–Alexa- Fluor-488 and/or goat anti-mouse IgG–Alexa-Fluor-546 (1:300, Molecular Probes) at room temperature. Images were captured using a Leica digital camera under a fluorescent microscope (Leica DMI6000B) or a Zeiss confocal microscope.

Primary antibodies against the following antigens were used: BrdU (1:50, Developmental Studies Hybridoma Bank at University of Iowa (DSHB)), Ki67 (1:500, Abcam), Pax6 (1:500, Covance), Pax6 (1:15 DSHB), Tbr2 (1:500, Abcam), GFP (1:1000, Abcam, chicken), and GFP (1:1000, Rockland, rabbit).

Cell counting in the mouse cortical tissue was performed in a fixed area of 300 μm × 300 μm, a representative column of the cortical wall from coronal sections. All sections analyzed were selected from a similar medial point on the anterior-posterior axis. For each condition, at least three brains, and at least three images for each individual brain were counted.

### Statistics

Data were shown as mean ± SEM. One-way analysis of variance (ANOVA) with *post hoc* contrasts were used for statistical analysis. The results were considered significant at probability level less than 0.05.

## Results

### miR-26 Is Co-expressed with Their Host Genes Ctdsp Throughout Cortical Development

**Figure 1:**
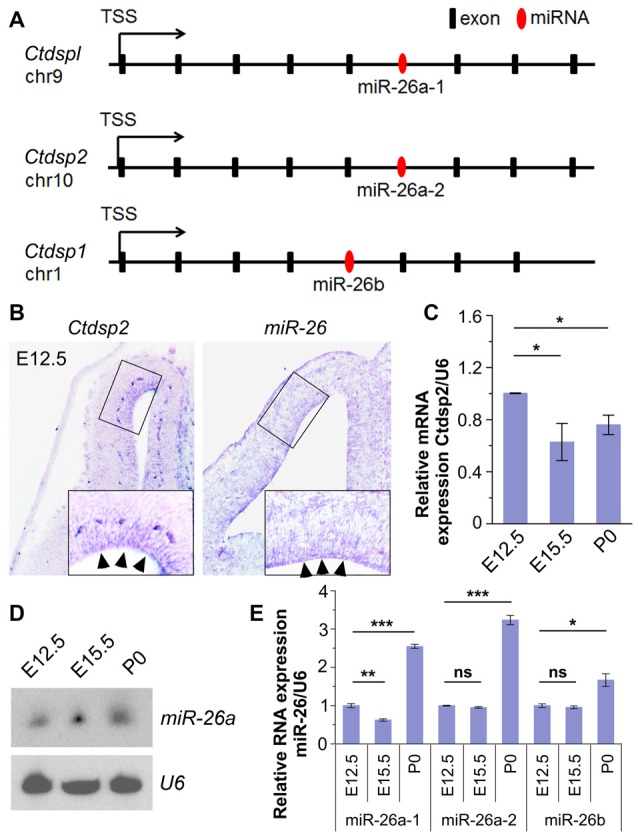
Expression patterns and levels of miR-26 and its host gene *Ctdsp2* in the mouse developing cortex. **(A)** Schemes of exons and introns of *Ctdspl*, *Ctdsp2* and *Ctdsp1*, and their intronic microRNA (miRNA) *miR-26a-1*, *miR-26a-2* and *miR-26b*, respectively, in the mouse genome. **(B)** *Ctdsp2* and *miR-26a* were both expressed in the ventricular zone (VZ; arrows). **(C)** Relative expression of *Ctdsp2* gene vs. U6 in E12.5, E15.5 and P0 cortices, detected by quantitative real-time PCR (qRT-PCR). *Ctdsp2* expression in the E12.5 cortex was set to 1. **(D)** *miR-26a* expression in different developmental stages, as detected by Northern blots. The ubiquitously expressed small non-coding RNA U6 was used as a loading control. **(E)** Relative expression levels of *miR-26a-1*, *miR-26a-2* and *miR-26b* in E12.5, E15.5 and P0 cortices, detected by qRT-PCR. *U6* was used as an internal control. miR-26 expression in the E12.5 cortex was set to 1. Values represent mean ± SEM. *n* > 3. *P < 0.05; **P < 0.01; ***P < 0.001; ns, not significant. Analysis of variance (ANOVA) with *post hoc* test was used.

To identify miRNAs that may function in neurogenesis, we performed expression profiles of miRNAs in the mouse cerebral cortex at embryonic day 12.5 (E12.5) and postnatal day 0 (P0) using microarray. The preliminary screen identified a miRNA, miR-26, which shows high expression at E12.5. Analyses of its seed sequence and genomic context indicated that the miR-26 family harbors two homologs, miR-26a and miR-26b, which are transcribed from three genomic loci, miR-26a-1, miR-26a-2 and miR-26b. Their seed sequences were highly conserved between species (Supplementary Figure S1A). Moreover, these loci reside in the introns of genes coding for *Ctdspl* in chromosome 9 (chr9), *Ctdsp2* in chr10 and *Ctdsp1* in chr1, respectively (Figure 1A).

We first examined whether miR-26a and its host gene Ctdsp2 are expressed concomitantly in the developing mouse cortices using *in situ* hybridization. The expression of *Ctdsp2* and *miR-26a* was mainly aggregated in the VZ in E12.5 cortex, indicating co-expression of miR-26 with its host gene Ctdsp2 (Figure 1B). Sense probe for *Ctdsp2* and scramble probe for *miR-26* did not show detectable signals (Supplementary Figure S1B). qRT-PCR validated that the expression of Ctdsp2 is high in the cortex at E12.5, slightly decreased from E12.5 to E15.5, and is merely altered from E15.5 to P0 (Figure 1C).

We next examined miR-26 expression. Northern blotting assay of miR-26a demonstrated that the expression of miR-26a is slightly up-regulated from E12.5 to E15.5, and is maintained at P0 (Figure 1D). The qRT-PCR assays of three miR-26 precursors further confirmed miR-26 expression throughout cortical development (Figure 1E). The expression of precursor miR-26a-1 was down-regulated from E12.5 to E15.5, and significantly increased at P0. Moreover, precursors miR-26a-2 and miR-26b displayed similar expression levels at E12.5 and E15.5, and were upregulated at P0 (Figure 1E). These data indicate that miR-26 and their host genes display opposite expression patterns during cortical development.

Due to the conserved seed sequence of miR-26 family, the following study was focused predominantly on the mouse miR-26a-2 host gene Ctdsp2, and miR-26a that contains the same seed sequence of both miR-26a-1 and miR-26a-2.

### Ctdsp2 Promotes Neural Progenitor Proliferation

**Figure 2:**
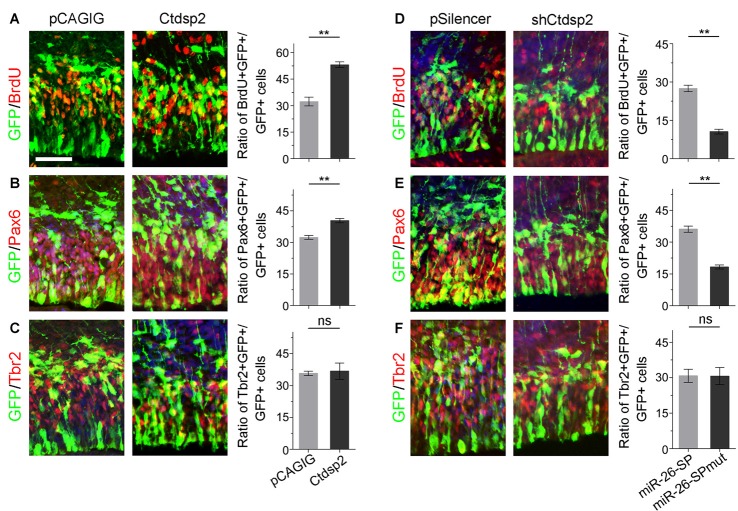
Ctdsp2 increases neural progenitor (NP) proliferation. **(A–C)** Overexpression of *Ctdsp2*, but not the control construct pCAGIG, increased the proportion of cells expressing both proliferative marker bromodeoxyuridine^+^ (BrdU^+^)/green fluorescence protein^+^ (GFP^+^) and radial glial cell (RGC) marker Pax6^+^/GFP^+^, but not intermediate progenitor (IP) marker Tbr2^+^/GFP^+^, in GFP-positive cells. **(D–F)** shRNA-mediated knockdown (sh*Ctdsp2*) of *Ctdsp2* decreased the proportion of both BrdU^+^/GFP^+^ cells and Pax6^+^/GFP^+^ cells, but not Tbr2^+^/GFP^+^ cells in GFP-positive cells, compared to the control construct pSilencer. Values represent mean ± SEM. *n* = 9 sections from at least three brains. **P < 0.01; ns, not significant. ANOVA with *post hoc* test was used. Scale bar = 50 μm.

Due to Ctdsp2 expression in the VZ of E12.5 mouse cortex, we examined the role of Ctdsp2 in regulation of NP development. We first overexpressed Ctdsp2 in NPs in the VZ using *in utero* electroporation at E13.5, and collected brain tissues at E14.5. A BrdU pulse was given 1 h before tissue collection to label dividing cells in the S-phase in a cell cycle. *Ctdsp2* overexpression, compared to the pCAGIG control construct, caused significant increase of the percentage of BrdU^+^/GFP^+^ cells vs. GFP^+^ cells, indicating that Ctdsp2 plays a role in promoting proliferation of cortical NPs (Figure 2A). We further investigated whether the population of RGCs and IPs, which can be labeled by Pax6 or Tbr2, respectively, is affected by Ctdsp2 (Englund et al., 2005). Ctdsp2 overexpression induced the expansion of Pax6^+^ RGCs, but did not change that of Tbr2^+^ IPs (Figures 2B,C).

We next knocked down *Ctdsp2* expression using short hairpin RNA (shRNA), shCtdsp2 (Supplementary Figure S2). Knockdown of *Ctdsp2* expression resulted in a significant reduction of the percentage of BrdU^+^/GFP^+^ cells vs. GFP^+^ cells, compared to the pSilencer control (Figure 2D). Moreover, shCtdsp2 significantly blocked the expansion of RGCs, and did not affect IPs (Figures 2E,F). These results indicate that Ctdsp2 promotes expansion of RGCs.

### miR-26a Positively Regulates Neural Progenitor Proliferation

**Figure 3:**
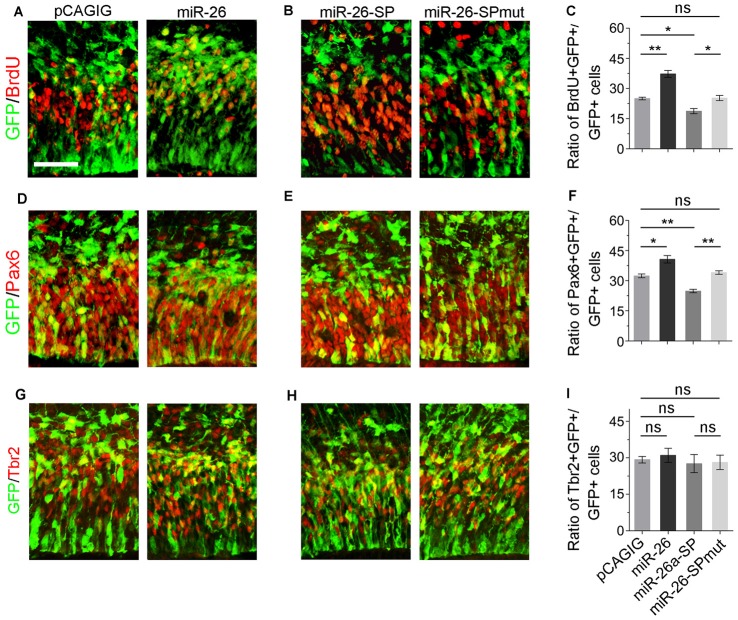
miR-26 promotes NP proliferation. **(A,D,G)** Overexpression of miR-26a, but not the control construct pCAGIG, proportion of cells expressing both proliferative marker BrdU^+^/GFP^+^ and RGC marker Pax6^+^/GFP^+^, but not IP marker Tbr2^+^/GFP^+^, in GFP-positive cells. **(B,E,H)** miRNA sponge-mediated knockdown (miR-26-SP), but not the mutated sponge (miR-26-SPmut), decreased proportion of cells expressing both proliferative marker BrdU^+^/GFP^+^ and RGC marker Pax6^+^/GFP^+^ in GFP-positive cells. **(C,F,I)** Ratio of BrdU^+^/GFP^+^, Pax6^+^/GFP^+^ or Tbr2^+^/GFP^+^ cells vs. GFP^+^ cells in the electroporated cortex. Values represent mean ± SEM. *n* = 9 sections from at least three brains. *P < 0.05; **P < 0.01; ns, not significant. ANOVA with *post hoc* test was used. Scale bar = 50 μm.

We next evaluated the role of miR-26a in NP development via overexpressing a construct containing the mouse miR-26a precursor using *in utero* electroporation. miR-26a overexpression significantly increased the percentage of BrdU^+^/GFP^+^ cells vs. GFP^+^ cells, suggesting that miR-26 facilitates NP proliferation (Figures 3A,C). Moreover, overexpression of miR-26a promoted the population of RGCs, and had no effect on regulating the population of IPs (Figures 3A,D,F,G,I).

To knock down miR-26a expression, we designed and applied miR-26 sponge (miR-26a-SP; Supplementary Figure S3; Otaegi et al., 2011). Opposite to miR-26a overexpression, miR-26a-SP led to a pronounced reduction of the percentage of BrdU+/GFP+ cells vs. GFP+ cells, while mutation of miR-26 sponge (miR-26-SPmut) showed no effect (Figures 3B,C). Moreover, knockdown of miR-26a decreased the population of RGCs, and did not change the population of IPs (Figures 3E,F,H,I). Our results suggest that proper expression levels of miR-26 are essential for mediating the proliferation of NPs and maintaining the size of the RGC population in the embryonic cortex.

### miR-26a Regulates the Cell-Cycle Progression of NPs

To further evaluate the mechanism of miR-26 function during neurogenesis, we analyzed its role in cell-cycle progression of NPs using *in utero* electroporation. Two parameters were quantified to indicate the status of cell-cycle. The labeling index was used to estimate the proportion of cells entering the cell cycle, by calculating BrdU incorporation in all cycling cells (Ki67^+^/GFP^+^). The cell-cycle-exit index was used to evaluate the proportion of cells exiting the cell cycle, by measuring the number of exiting cells within 24 h of BrdU incorporation (BrdU^+^/Ki67^−^/GFP^+^) vs. the total number of cycling cells (Ki67^+^/GFP^+^).

**Figure 4:**
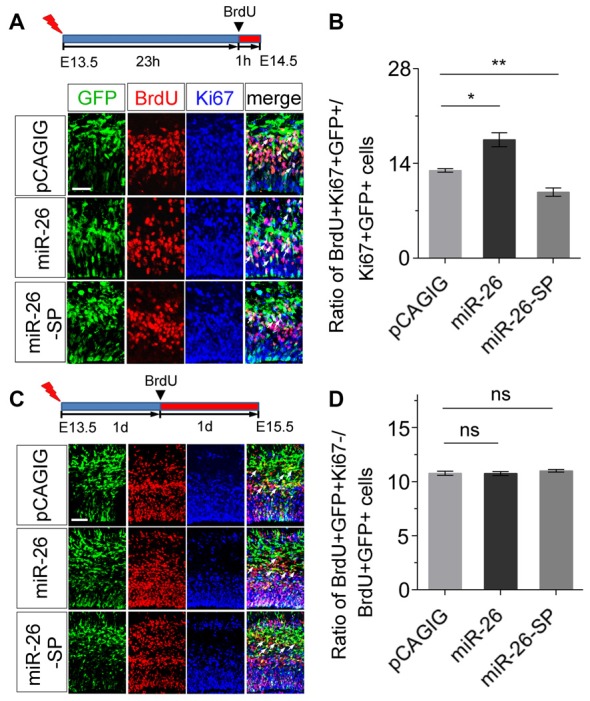
miR-26a regulates cell cycle progression in NPs. **(A)** Illustration of *in utero* electroporation and BrdU incorporation. Electroporation was performed at E13.5, followed by BrdU incorporation 23 hours (23 h) later, and tissues were analyzed at E14.5. BrdU^+^, Ki67^+^ and GFP^+^ cells were labeled and counted. **(B)** miR-26a overexpression increased the proportion of cells entering the S phase of the cell cycle, while miR-26a-SP decreased the proportion, by measuring the BrdU labeling index. **(C)** Electroporation was performed at E13.5, followed by BrdU incorporation 1 day (1d) later, and tissues were analyzed at E15.5. BrdU^+^, Ki67^+^ and GFP^+^ cells were labeled and counted. **(D)** Neither miR-26a nor miR-26-SP altered the proportion of cells exiting the cell cycle. Values represent mean ± SEM. *n* = 9 sections from at least three brains. *P < 0.05; **P < 0.01; ns, not significant. ANOVA with *post hoc* test was used. Scale bar = 50 μm.

To analyze the labeling index, the embryonic cortex was electroporated at E13.5, and collected at E14.5. BrdU was injected 1 h before brain tissue collection (Figure 4A). miR-26a overexpression caused increased proportion of BrdU^+^/Ki67^+^ cells vs. GFP^+^ cells, compared to the control (Figures 4A,B). On the contrary, miR-26a knockdown using its sponge displayed a reduction of BrdU^+^/Ki67^+^ cells vs. GFP^+^ cells (Figures 4A,B). The cell-cycle labeling index analysis suggests that miR-26a promotes NPs reenter the cell cycle.

To analyze the cell-cycle exit, the embryonic cortex was electroporated at E13.5, and collected at E15.5. BrdU was injected 1 day before brain tissue collection (Figure 4C). Neither miR-26a overexpression nor knockdown resulted in significant changes on the ratio of BrdU^+^/Ki67^−^ cells vs. GFP^+^ cells, suggesting that miR-26a is not involved in the regulation of cell-cycle exit during the G1/S transition in NPs (Figures 4C,D).

### Identification of Emx2 as a Target for miR-26a in NP Proliferation

**Figure 5:**
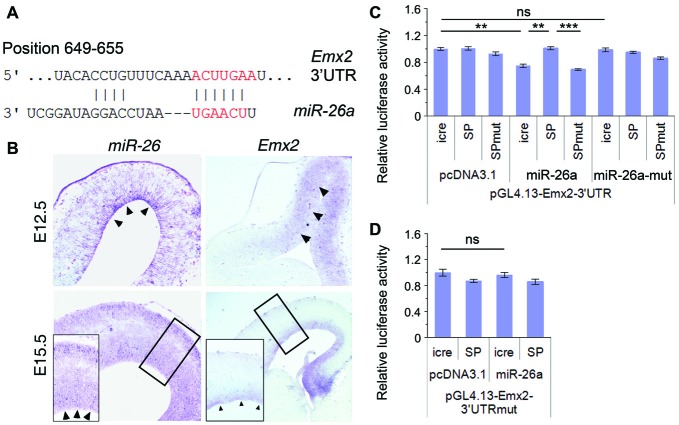
Emx2 is a target of miR-26a. **(A)** *Emx2* 3′ untranslated region (3′UTR) contains a binding site for miR-26. The seed sequence is shown in red. **(B)** miR-26 and *Emx2* were co-expressed in the VZ and subventricular zone (SVZ; arrowheads) in E12.5 and E15.5 cortices, detected by *in situ* hybridization. Inset images show high power views of highlighted regions in E15.5 cortices. **(C)** miR-26a suppressed luciferase activities in the construct containing the *Emx2–*3′UTR, while miR-26-SP but not miR-26a-SPmut rescued the suppression. miR-26a-mut had no suppressing activity. **(D)** The mutation of miR-26 binding sites in the *Emx2–*3′UTR displayed no silencing activity by miR-26. *n* > 3. **P < 0.01; ***P < 0.001; ns, not significant. ANOVA with *post hoc* test was used.

miRNAs generally function via silencing coding genes. Our bioinformatic analysis predicted Emx2 (empty spiracles homeobox 2), which contains a binding site for miR-26a at the 3′UTR, as a potential targeted gene for miR-26 (Figure 5A). Previous studies suggest that miRNAs often have overlapping expression with their target genes in specific tissues (Hobert, 2007; Karres et al., 2007). We thus compared expression patterns of miR-26 and Emx2 in E12.5 and E15.5 mouse cortices using *in situ* hybridization. miR-26 expression was detected in the VZ in E12.5 cortices, and its expression in the VZ/SVZ was decreased in E15.5 cortices. Compared to miR-26a, Emx2 expression was observed in the VZ and SVZ in both E12.5 and E15.5 cortices with a higher and broader expression compared to that of miR-26, indicating overlapping but also distinct expression of miR-26 and Emx2 (Figure 5B).

To verify miR-26 targeting effect on Emx2, we performed luciferase assays by testing the *Emx2* 3′UTR and its mutation where miR-26 binding sites have been mutated. The luciferase activity in constructs containing the *Emx2* 3′UTR was notably repressed by miR-26a but not by miR-26 mutation (miR-26a-mut). Moreover, miR-26 sponge (miR-26-SP) could rescue miR-26 silencing effect on the *Emx2* 3′UTR, its mutation (miR-26-SPmut) showed no effect on the *Emx2* 3′UTR (Figure 5C). To further test the specificity of miR-26 on silencing the *Emx2* 3′UTR, we generated a mutant form in which the miR-26 binding sites in the *Emx2* 3′UTR are mutated, named *Emx2* 3′UTRmut. When it was co-expressed with miR-26, the luciferase activity did not show significant reduction, indicating that miR-26a failed to silence *Emx2* 3′UTRmut (Figure 5D). These results suggest that Emx2 is a specific putative target for miR-26a.

### Cohesive Regulatory Roles of miR-26a and Its Target Emx2 in NP Proliferation

**Figure 6:**
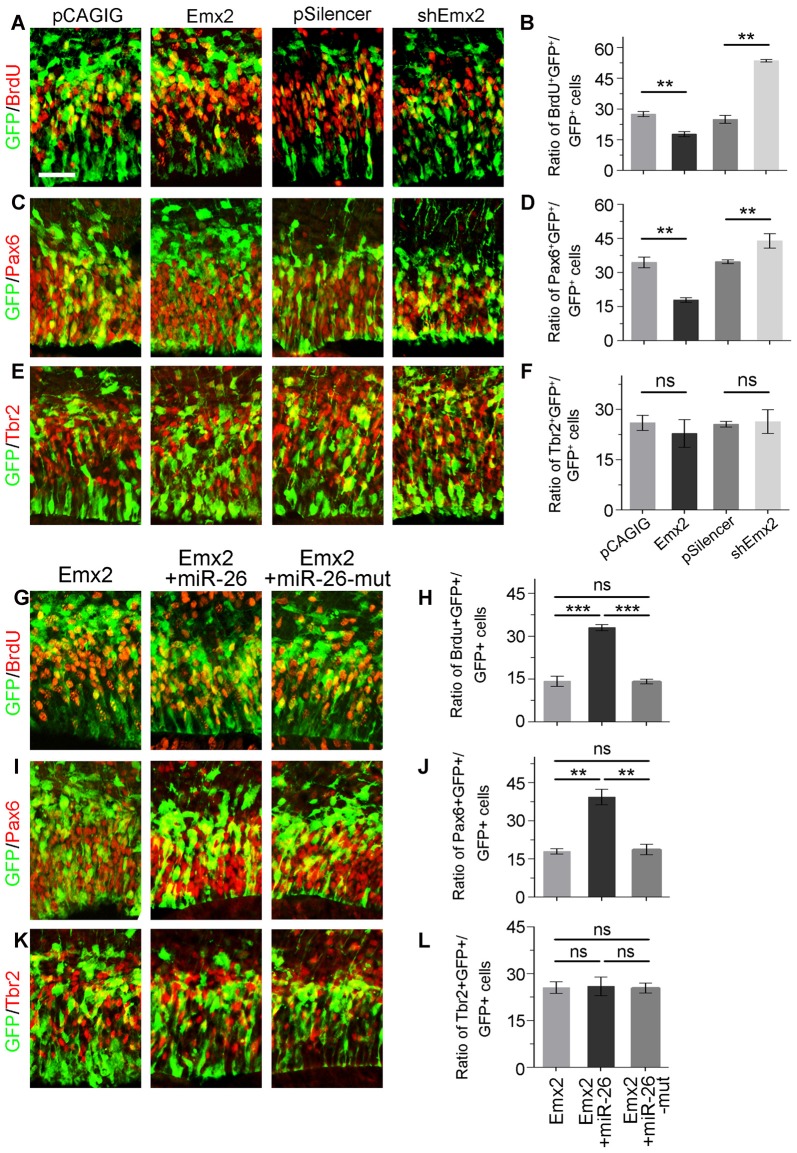
Emx2 is functionally inhibited by miR-26 in regulating NP proliferation. **(A–F)** Overexpression of *Emx2*, but not the control construct pCAGIG, decreased the proportion of cells expressing both proliferative marker BrdU^+^/GFP^+^ and RGC marker Pax6^+^/GFP^+^, but not IP marker Tbr2^+^/GFP^+^, in GFP-positive cells. short hairpin RNA (shRNA)-mediated knockdown (sh*Emx2*) of *Emx2* increased the proportion of both BrdU^+^/GFP^+^ cells and Pax6^+^/GFP^+^ cells, but not Tbr2^+^/GFP^+^ cells in GFP-positive cells, compared to the control construct pSilencer. **(G–J)** Emx2 expression suppressed the proportion of cells expressing both proliferative marker BrdU^+^/GFP^+^ and RGC marker Pax6^+^/GFP^+^ in GFP-positive cells. Co-expressing Emx2 with miR-26, but not miR-26-mut, dramatically reversed the suppression. **(K,L)** Emx2 expression did not alter the proportion of cells expressing IP marker Tbr2^+^/GFP^+^, in GFP-positive cells. Values represent mean ± SEM. *n* = 9 sections from at least three brains. **P < 0.01; ***P < 0.001; ns, not significant. ANOVA with *post hoc* test was used. Scale bar = 50 μm.

Due to the overlapping expression of miR-26a and its target Emx2 in the VZ and SVZ in the cortex, we examined whether Emx2 plays a similar or opposite role in NP development by overexpressing and silencing Emx2 at E13.5 (Supplementary Figure S2). Brain tissues were collected 24 h after *in utero* electroporation. Overexpression of Emx2, compared to the control, significantly decreased the percentage of BrdU^+^/GFP^+^ cells vs. GFP^+^ cells (Figures 6A,B). The population of Pax6^+^ RGCs was also decreased (Figures 6C,D). Conversely, knockdown of Emx2 using shEmx2 greatly increased the percentage of BrdU^+^/GFP^+^ NPs and RGCs (Figures 6A–D). Altered Emx2 expression did not cause detectable changes of the IP population (Figures 6E,F). Our results suggest that Emx2 plays a role in suppressing expansion of cortical NPs.

To further investigate the targeting interaction between miR-26a and Emx2 in NP development, miR-26a was co-electroporated with Emx2 full length cDNA that contains its 3′UTR in the E13.5 mouse cortex. Compared to Emx2 alone, co-electroporation of Emx2 with miR-26 caused a pronouncing elevation of the population of BrdU^+^/GFP^+^ cells vs. GFP^+^ cells and the population of Pax6^+^/GFP^+^ cells vs. GFP^+^ cells (Figures 6G,H). However, co-electroporation of Emx2 with miR-26 mutation in the seed sequence (miR-26-mut) did not change the proportion of BrdU^+^/GFP^+^ or Pax6^+^/GFP^+^ cells (Figures 6I,J). These results suggest that miR-26 specifically blocks the function of Emx2 in suppressing NP proliferation. Additionally, co-expression of Emx2 and miR-26 or miR-26-mut did not alter the proportion of Tbr2^+^/GFP^+^ cell populations, suggesting that Emx2 and miR-26 mostly regulate the RGC but not IP population (Figures 6K,L). Our results suggest a cohesive regulatory role of miR-26a and Emx2 in cortical NP expansion.

### Emx2 Binding Activities to Promote miR-26 Host Genes Ctdsp

**Figure 7:**
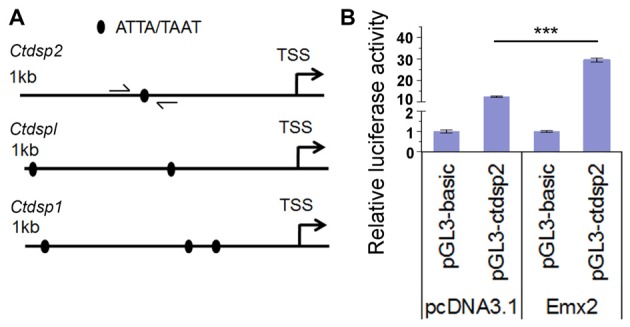
Emx2 promotes Ctdsp2 transcription. **(A)** The 1 kb upstream regions of the transcription start site (TSS) of *Ctdsp* genes contained the Emx2 binding sites. **(B)** Co-expressing Emx2 with 1 kb promoter of Ctdsp2 increased luciferase activities. *n* > 3, ***P < 0.001. ANOVA with *post hoc* test was used.

Several studies have shown that target genes of some miRNAs have a feedback regulation on miRNA expression (Bian and Sun, 2011). To investigate whether Emx2 might regulate expression of miR-26 host genes *Ctdsp*, we searched the promoter regions of three *Ctdsp* genes. Previous work has identified ATTA or TAAT as a binding motif for the transcription factor Emx2 (Mariani et al., 2012). Interestingly, 1 kb upstream regions of the transcription start site (TSS) of all three *Ctdsp* genes contained 1–3 Emx2 binding sites, suggesting that Emx2 may regulate *Ctdsp* expression, subsequently miR-26 expression, by binding to the *Ctdsp* promoter regions (Figure 7A). To further text Emx2 binding activities, 1 kb and 2 kb promoters of *Ctdsp2* were cloned into a luciferase reporter vector, and were co-expressed with Emx2. The luciferase activities were significantly increased when Emx2 was co-expressed, compared to the control (Figure 7B). These results suggest that Emx2 may function as a transcription activator on *Ctdsp* gene expression and initiates miR-26 expression.

## Discussion

Dysregulation of proliferation, survival and differentiation of NPs causes malformation of cortical architecture, in turn the brain dysfunction, for example epilepsy and mental retardation (Molnár, 2011; Sun and Hevner, 2014). Revealing the regulatory mechanism of cortical development will contribute to a deep understanding of brain disorders and possible clinical therapies. The emerging evidence highlights critical roles of miRNAs in brain development and disorders (Livak and Schmittgen, 2001; Mellios and Sur, 2012; Mellios et al., 2017). In this study, we demonstrate that miR-26a, its target Emx2 and its host gene *Ctdsp2* cohesively mediate NP proliferation and RGC expansion using a loop-regulatory mechanism during the mouse cortical development.

Proper development of the cortex relies on the proliferation and expansion of NPs, including RGCs and IPs, which are tightly regulated by precise expression patterns of both coding and noncoding RNAs (Rakic, 2009; Sun and Shi, 2015). Ctdsp2, also known as SCP2, is a phosphatase involved in regulating both signaling pathways and gene transcription (Yeo et al., 2003; Knockaert et al., 2006; Sapkota et al., 2006). Studies have shown that Ctdsp2 induces cell-cycle arrest and decreases the number of cells in S-phase in both human and mouse embryonic cells (Kashuba et al., 2004; Zhu et al., 2012; Kloet et al., 2015). Interestingly, our studies have shown an opposite role of Ctdsp2 in regulating the cell cycle to facilitate proliferation of NPs and expansion of RGCs in the mouse embryonic cortex. One possible reason for the conversed function of Ctdsp2 is likely due to an overlapping co-expression of its intronic miRNA miR-26. Studies have shown that most host genes are functionally associated with their intronic miRNAs (Woltering and Durston, 2008; Lund, 2010; Small et al., 2010). Expression of Ctdsp2 may concomitantly induce upregulation of miR-26 transcript level in the mouse cortex, and in turn promote NP proliferation.

Furthermore, miR-26 was previously reported to promote neuronal differentiation by suppressing *Ctdsp* in zebrafish (Dill et al., 2012; Han et al., 2012). Our studies have shown that miR-26-dependant regulation of the cell-cycle progression determines reentering but not exiting the cell-cycle in the NP population, which suggests a positive regulatory role in NP proliferation and expansion. These data indicate distinct roles of miR-26 between two species. Moreover, miR-26 can be either an activator or a suppressor of cell proliferation in different cell types in even the same species (Gao and Liu, 2011). Studies on miR-26 have also shown its inhibitory role in proliferation, migration and differentiation via targeting PFKFB3, EZH2 and other downstream genes (Lu et al., 2011; Du et al., 2015; Chen et al., 2016), and a positive role by targeting GSK-3beta, PTEN CHD1 and downstream genes (Huse et al., 2009; Kim et al., 2010; Zhang et al., 2012; Tan et al., 2014). Distinct functions of miR-26 are likely achieved through regulating different target genes in different cell types.

In the mouse cerebral cortex, we have identified miR-26a target gene Emx2, which functions in an opposite manner to miR-26a in regulating NP proliferation and RGC expansion. Studies have shown that Emx2 functionally interacts with specific transcript factors, like Sox2 and Pax6 in the developing cortex (Muzio et al., 2002a,b; Mariani et al., 2012). Notably, Emx2 cooperates with Pax6 in regulating neocortex development (Bishop et al., 2000; Bayatti et al., 2008). Emx2 regulates transcription levels of downstream genes associated with neuronal proliferation (Gangemi et al., 2006). Moreover, a report in *Paralichthys olivaceus* also indicated Emx2 as a target gene of miR-26a and miR-26b (Yin et al., 2015). In this study, we have demonstrated that Emx2 is a potential activator to facilitate the expression of miR-26 host gene *Ctdsp*, and subsequently the expression of its intronic miRNA miR-26.

Altogether, the concomitant expressions of miR-26 and their host genes *Ctdsp* play a positive role in NP proliferation and RGC expansion in the developing cortex. On the other hand, miR-26 target gene Emx2 negatively controls NP development and activates expression of *Ctdsp*, and further initiates miR-26 transcription, although the directly or indirectly regulatory function between Emx2 and Ctdsp requires further exploration in biological meanings. It is likely that miR-26 and its target Emx2 form a regulatory-loop via miR-26 host gene Ctdsp as an intermediator, in maintaining proper populations of NPs and RGCs during the cortical development. Our findings provide a new insight into the regulatory mechanism of miRNA miR-26-mediated NP development and cortical formation.

## Author Contributions

HZ and LZ contributed equally to this work. TS supervised the process of this work.

## Conflict of Interest Statement

The authors declare that the research was conducted in the absence of any commercial or financial relationships that could be construed as a potential conflict of interest.
