# Supplementary material for: Cohesive Regulation of Neural Progenitor Development by microRNA miR-26, Its Host Gene Ctdsp and Target Gene Emx2 in the Mouse Embryonic Cerebral Cortex
Source: Front Mol Neurosci. 2018 Feb 19;11:44. doi: 10.3389/fnmol.2018.00044 (PMC5825903; doi:10.3389/fnmol.2018.00044)
Supplement: Supplementary file 1 [file Presentation_1.pdf]

Cohesive regulation of neural progenitor development by microRNA miR-26, its host gene Ctdsp and target gene Emx2 in the mouse embryonic cerebral cortex

Haijun Zhang[1,*], Longbin Zhang[2,*], and Tao Sun[1,2,#]

[1]Department of Cell and Developmental Biology, Cornell University Weill Medical College, 1300 York Avenue, New York, NY 10065

[2]School of Medicine and School of Biomedical Sciences, Huaqiao University, Quanzhou, Fujian, China 361021

[#]Corresponding author: Dr. Tao Sun, Email: taosun@hqu.edu.cn

*These authors contributed equally to this work.

Running head: Regulatory loop of miR-26

Keywords: miR-26, Ctdsp, Emx2, neural progenitor, cell-cycle progression

Supplementary figure legends

FIGURE S1. (A) Conserved mature sequences of miR-26 among species. Comparison of each miR-26 homolog sequence in *Mus musculus* (mmu), *Homo sapiens* (has) and *Danio rerio* (dre). The seed sequence is shown in blue, and mismatched nucleotide is shown in red. **(B)** The *in situ* control staining for Ctdsp2 with sense probe, miR-26 with control probe and Emx2 with sense probe respectively.

FIGURE S2. Relative expression of Ctdsp2 and Emx2 in cells carrying pSelincer vector versus shRNA-expressing vector. Values represent mean ± SEM, n>3. * $P < 0.05$; *** $P < 0.001$. Analysis of variance (ANOVA) with post hoc test was used.

FIGURE S3. Design of miR-26 sponge. miR-26 sponge and its mutated sequence compared to three miR-26 homologs. The seed sequence is shown in blue, mismatched nucleotide is shown in red and the mutated nucleotide is shown in green.

FIGURE S1

A
```
mmu-miR-26a-1  5'  UUCAAGUAAUCCAGGAUAGGCU  3'
hsa-miR-26a-1  5'  UUCAAGUAAUCCAGGAUAGGCU  3'
dre-miR-26a-1  5'  UUCAAGUAAUCCAGGAUAGGCU  3'

mmu-miR-26a-2  5'  UUCAAGUAAUCCAGGAUAGGCU  3'
hsa-miR-26a-2  5'  UUCAAGUAAUCCAGGAUAGGCU  3'
dre-miR-26a-2  5'  UUCAAGUAAUCCAGGAUAGGCU  3'

dre-miR-26a-3  5'  UUCAAGUAAUCCAGGAUAGGCU   3'

mmu-miR-26b    5'  UUCAAGUAAUUCAGGAUAGG-U  3'
hsa-miR-26b    5'  UUCAAGUAAUUCAGGAUAGG-U  3'
dre-miR-26b    5'  UUCAAGUAAUCCAGGAUAGGUU  3'
```

seed sequence
Mismatch
miRNA is named based on its stem-loop sequence

B

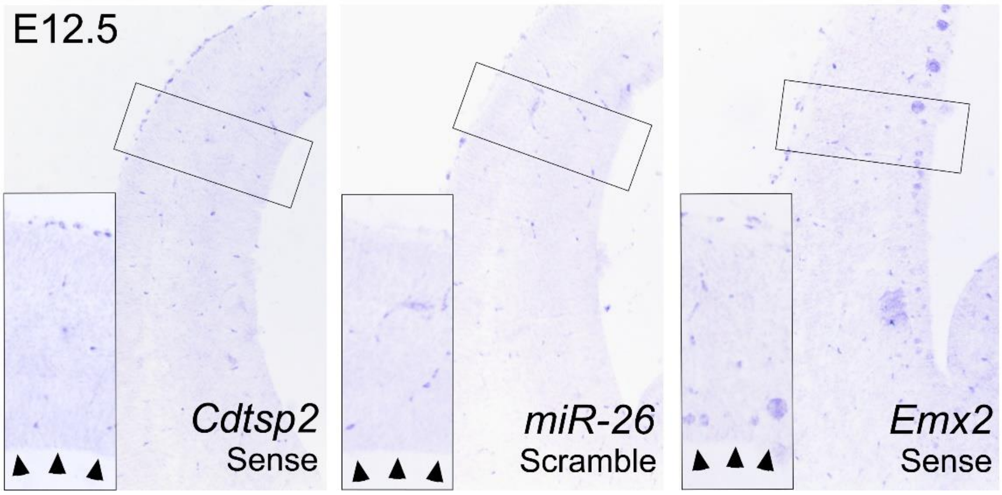

FIGURE S2

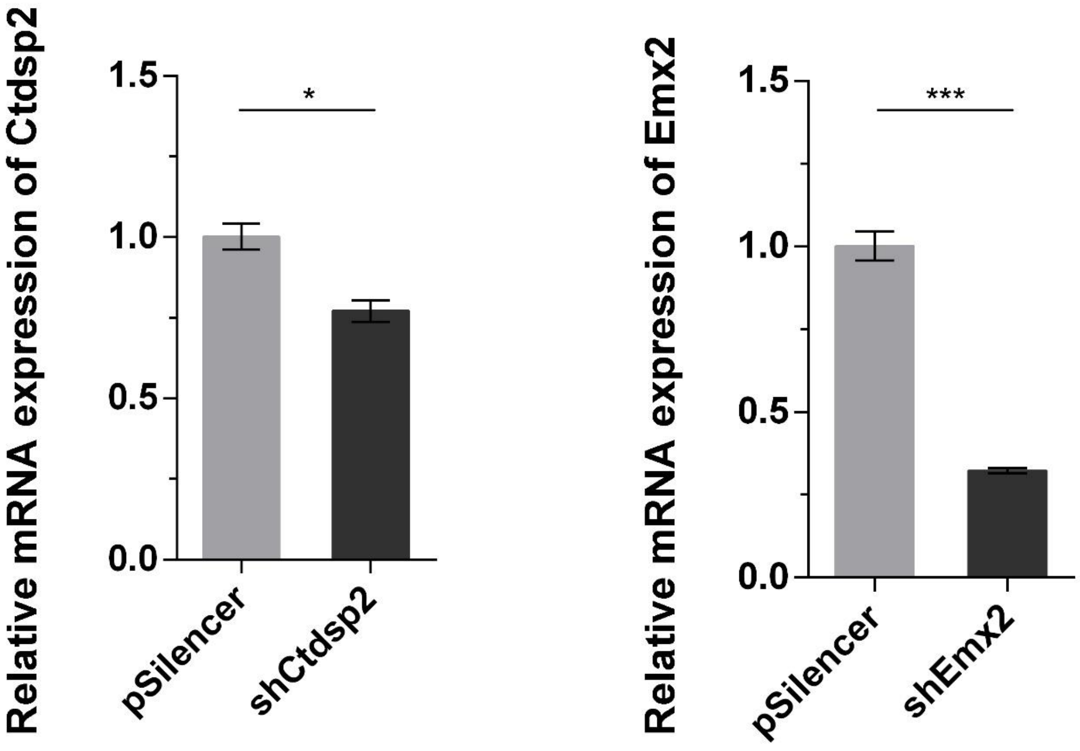
Relative mRNA expression of Ctdsp2
1.5
1.0
0.5
0.0
*
pSilencer
shCtdsp2
Relative mRNA expression of Emx2
1.5
1.0
0.5
0.0
***
pSilencer
shEmx2

FIGURE S3

```
26SP unit     5′ AGCCUAUCCUGCU-UACUUGAA 3′
26SPmut unit  5′ AGCCUAUCCUGCU-UAGUUCUA 3′
miR-26a-1     3′ UCGGAUAGGACCUAAUGAACUU 5′
miR-26a-2     3′ UCGGAUAGGACCUAAUGAACUU 5′
miR-26b       3′ U-GGAUAGGACUUAAUGAACUU 5′
```

seed sequence

Mismatch

Mutated nucleotide
